# Gas-like adhesion of two-dimensional materials onto solid surfaces

**DOI:** 10.1038/s41598-017-00184-x

**Published:** 2017-03-13

**Authors:** Zhengrong Guo, Tienchong Chang, Xingming Guo, Huajian Gao

**Affiliations:** 1 Shanghai Institute of Applied Mathematics and Mechanics, Shanghai Key Laboratory of Mechanics in Energy Engineering, Shanghai University, Shanghai, 200072 People’s Republic of China; 2 State Key Laboratory of Ocean Engineering, School of Naval Architecture, Ocean and Civil Engineering, Shanghai Jiao Tong University, Shanghai, 200240 People’s Republic of China; 3 School of Engineering, Brown University, Providence, Rhode, Island 02912 USA

## Abstract

The adhesion of two-dimensional (2D) materials onto other surfaces is usually considered a solid-solid mechanical contact. Here, we conduct both atomistic simulations and theoretical modeling to show that there in fact exists an energy conversion between heat and mechanical work in the attachment/detachment of two-dimensional materials on/off solid surfaces, indicating two-dimensional materials adhesion is a gas-like adsorption rather than a pure solid-solid mechanical adhesion. We reveal that the underlying mechanism of this intriguing gas-like adhesion is the configurational entropy difference between the freestanding and adhered states of the two-dimensional materials. Both the theoretical modeling and atomistic simulations predict that the adhesion induced entropy difference increases with increasing adhesion energy and decreasing equilibrium binding distance. Our findings provide a fundamental understanding of the adhesion of two-dimensional materials, which is important for designing two-dimensional materials based devices and may have general implications for nanoscale efficient actuators.

## Introduction

Adhesion between surfaces is one of the most common and important phenomena in nature. As a surface phenomenon, the influence of adhesion increases with decreasing the size of contacting materials due to the increase of the surface-to-volume ratio. In particular, at the nanoscale, where nanomaterials have ultra-high surface-to-volume ratio, adhesion becomes extremely strong and dominant in many processes related to the synthesis, transfer and device integration of nanomaterials^1^. However, due to the emerging of scale effects and the lack of knowledge of related physics, adhesion of nanomaterials onto other materials or surfaces remains to be understood.

Recent studies have focused on the interaction between two-dimensional (2D) materials and solid surfaces^2–7^. Two-dimensional materials can conform more closely to a surface than other materials^8^, which is significantly different from bulk solid–solid adhesion^9^. In addition, 2D materials usually exhibit strong thermal activity even at extremely low temperatures due to their atomically thin structures^10^. However, in existing theoretical studies, 2D materials are usually considered as mechanical sheets and their adhesion to other surfaces is treated as a mechanical contact. In fact, thermal or temperature-dependent adhesion phenomena of 2D materials have been observed in some works^11–14^. For example, the intrawall adhesion can collapse a large-diameter carbon nanotube (which can be viewed as a cylindrical graphene layer) into a flat configuration, while at some higher temperature the collapsed tube can restore its cylindrical shape^12, 13^. Without a better understand of the role of thermal effect on 2D materials adhesion, such phenomena cannot be well understood in a pure mechanical way.

Here, we report a new adhesion mechanism of 2D materials onto solid surfaces, revealing that the adhesion is not solely a mechanical adhesion but also a gas adsorption. Our results indicate that both the adhesion energy and adhesion forces are not constants solely determined by physical interaction forces. In contrast, they depend also on system temperatures. Importantly, there exists a nearly reversible energy conversion between thermal energy and mechanical work in the attachment/detachment of a 2D material on/off a surface. We present an analytical model based on phonon analysis to elucidate the underlying physics of this intriguing gas-like adhesion.

**Figure 1:**
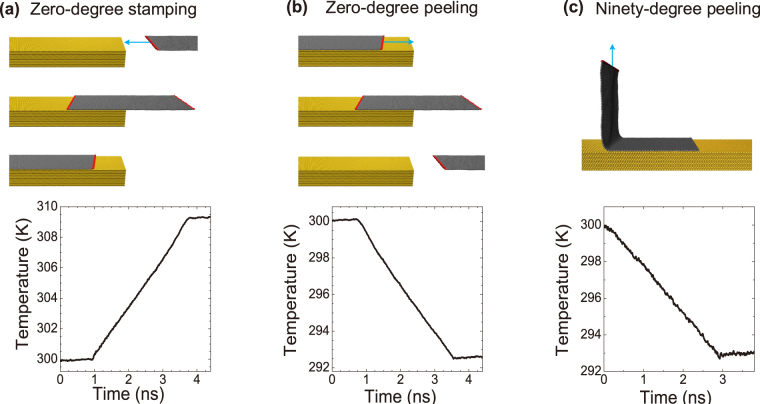
Temperature change of a graphene ribbon during the adiabatic processes of zero-degree stamping (**a**), zero-degree peeling (**b**) and ninety-degree peeling (**c**) of the graphene ribbon on/off the (111) surface of a Pt substrate. The edges (in red) of the graphene ribbon are set to be rigid where a constant velocity of 10 nm/ns is imposed to move the graphene ribbon to attach onto or detach off the substrate. The temperature at time *t* is calculated by averaging through a small period of 1 ps around *t*.

To gain some insights into the adhesion mechanisms of 2D materials, we first perform two types of atomistic simulations of adiabatically stamping/peeling of a graphene ribbon on/off the (111) surface of a platinum (Pt) substrate. We use the second generation reactive empirical bond order (REBO)^15^ to describe the C-C bond interaction in graphene and Lennard-Jones 12-6 potential to describe the interaction between the graphene layer and the substrate (details can be found in the Supplemental Material). We simulate zero-degree stamping and peeling of a graphene ribbon as illustrated in Fig. 1(a) and (b). During the simulations, the graphene ribbon is moved onto or away from the substrate by a constant velocity imposed on the two rigid edges. The Pt substrate is set to be rigid and the temperature change in the graphene layer induced by heat release and extraction is carefully monitored.

Figure 1(a) and (b) show the temperature changes of the graphene ribbon during the adiabatically stamping and peeling processes, respectively, which take place for 2.85 ns out of a total simulation time of 4.5 ns in both cases. Significant temperature variations show thermodynamic tendencies for heat release during stamping and heat absorption during the peeling. To further confirm the inherence of heat release/absorption in the attachment/detachment of the graphene, we simulate the peeling of a graphene ribbon at ninety degrees from a Pt substrate, where a similar temperature change is observed (Fig. 1c). In all three simulations, no other energy forms beside thermal energy and mechanical energy (including peeling work and the van der Waals energy between the layer and the substrate) are involved. Thus the heat release in the stamping process is converted from mechanical energy, and the heat absorption in a peeling process is converted to mechanical work. The magnitude of the temperature changes in the first two cases are quite close, showing the energy conversions in stamping processes and peeling processes are nearly thermodynamically reversible from one state to the other. It also indicates that friction between the graphene ribbon and the smooth (111) surface of the Pt substrate^16, 17^ in our simulations is insignificant. The low friction in the simulations also explains why the temperature is nearly a constant during the graphene is being totally adhered and sliding on the substrate (the first 0.9 ns in the Fig. 1a and the last 0.9 ns in Fig. 1b) where the friction induced dissipation should reach a maximum due to the maximum contacting area^18^.

Although physical adhesion between bulk solid materials may induce heat release in an attachment process due to deformation and friction, it is impossible to give rise to heat adsorption in detachment. In contrast, gas adsorption on solid surface can release heat and gas desorption form solid surface can absorb heat^19^. In this respect, 2D materials adhesion is gas-like. The similarity of materials nature between gas and 2D materials seems to be responsible for their similar thermodynamic adhesion behaviors. When gas molecules are adhered on a surface, they form a 2D film that closely conforms to the surface, while 2D materials also can closely conform to a surface due to their extremely low bending rigidity^6, 10, 20^. As a result, thermal activities are significant in the 2D materials adhesion, leading to a significant difference from a mechanical adhesion between bulk solids.

**Figure 2:**
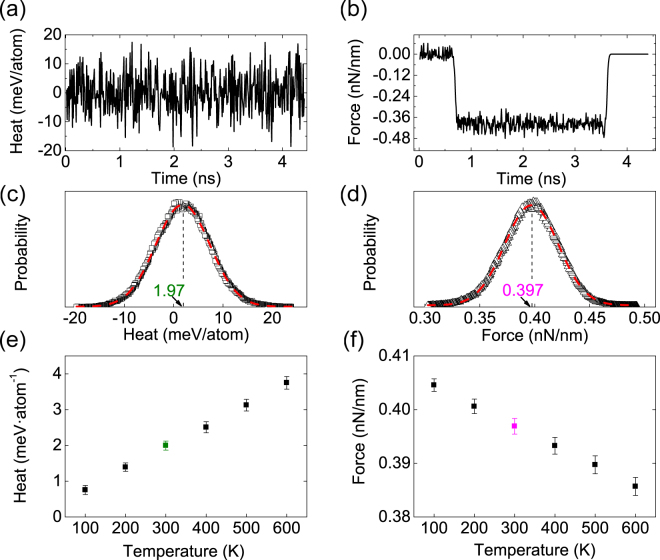
Isothermal simulations (100–600 K) of a graphene ribbon peeled from a rigid Pt substrate at zero-degree. (**a**) Thermal energy exchange between the graphene ribbon and the thermostat (300 K). (**b**) Shear adhesion force (per nanometer in width) on the graphene ribbon (300 K). (**c**) Distribution of the heat exchanges (300 K) during the detachment (from 0.9 ns to 3.6 ns). Red dash line is the Gauss fitting of the MD results. (**d**) Distribution of the shear adhesion force (300 K) during the detachment. (**e**) Isothermal heat absorptions of the graphene ribbon at different temperatures. (**f**) The shear adhesion force versus system temperature.

The underlying physics of gas-like adhesion of 2D materials is that the configurational entropy of a freestanding 2D layer is larger than that of an adhered one. The mechanism can be attributed to a drop in configurational entropy as the substrate confines the out-of-plane fluctuations of an adhered 2D material layer and reduces its degrees of freedom. To confirm the entropy change due to adhesion, we calculated the stamping and peeling of graphene under isothermal conditions, following the universal approach used to describe gas adsorptions^19^. A Berendsen thermostat is used to keep the 2D materials at constant temperatures during the attachment and detachment, and the isothermal heat releases/absorptions are measured from the heat exchanges between the thermostat and the 2D materials. The heat release or absorption induced by the adhesion caused configurational entropy change Δ*S* in a reversible process under isothermal conditions can be theoretically expressed as Δ*E* = −*T* · Δ*S*, with *T* being the temperature. This linear dependence of heat change on temperature is confirmed in our MD simulations, as shown in Fig. 2e.

The configurational entropy changes are usually accompanied by a phenomenologically entropic force. Thus as a result, the adhesion force between a 2D layer and a substrate should consist two parts,

1 $$F={F}_{{\rm{vdw}}}+{F}_{{\rm{entr}}},$$

where the first term *F*_vdw_ = ∇_*x*_ *E*_vdw_ (in which ∇_*x*_ is the differential operator with respect to the path variable *x*, *E*_vdw_ is the interlayer van der Waals potential) is the van der Waals force, and the second term is the entropic force given by *F*_entr_ = −*T*∇_*x*_ *S*. At a zero-degree peeling, the adhesion force is indeed the interlayer shear force. To validate Eq. (1), we plot the shear adhesion force of graphene on Pt substrate from MD simulations as functions of the system temperature in Fig. 2f. It clearly shows that the shear force is linearly dependent on temperature, which can be safely attributed to entropic force because the interlayer van der Waals force is a constant.

In the experimental research field of 2D materials adhesion, a number of approaches were developed to determine the adhesion energy on various substrates^2, 3, 7^. These approaches measure the adhesion force through a force balance between an external loading force and the adhesion force between a 2D layer and a substrate^9^. Typically, at a zero-degree peeling test^7^, the critical peeling force, which is equal to the shear adhesion force at balance, is measured and used to estimate the adhesion energy. However, as we have shown, the adhesion force consists not only a conservative van der Waals force but also a temperature-dependent entropic force, which would result in a fundamental difference between the experimentally measured and the theoretically predicted *E*_vdw_^21, 22^. The ratio of the adhesion force *F*_entr_ to the van der Waals force *F*_vdw_ is solely dependent on temperature, and is independent of the path variable *x* as in Eq. (1), indicating this difference is a constant at a given temperature and increases linearly with temperature. In our simulations, at room temperature, this difference is found to be about 3% for the adhesion of graphene on a Pt substrate, and 7% for the adhesion between two graphene layers.

Now we intend to elucidate how adhesion causes a configurational entropy change in 2D materials. In fact, the entropy change due to external confinement has been extensively studied in many phenomena^23–25^. We analyze the adhesion induced configurational entropy change based on the analysis of normal modes, each of which representing a harmonic mode of vibration of all atoms in a 2D material at a certain frequency. The adhesion is described by a potential well perpendicular to the surface. Under this confining potential, it can be shown that the frequencies of the out-of-plane normal modes are shifted to higher values^26^

2 $${\omega }_{{\rm{ad}}}=\sqrt{{\omega }^{2}+\kappa /m}$$

where *m* is the atomic mass of the 2D material and *κ* is the curvature of the confining potential. A quantum statistical analysis of the normal modes indicates that the increase in frequency results in a decrease in configurational entropy, as a result of reduced number of microstates (see Supplemental Materials for details). Finally, we find that the adhesion induced entropy change of a 2D material can be expressed as

3 $${\rm{\Delta }}S=\frac{1}{2}{{\rm{k}}}_{{\rm{b}}}\,[\mathrm{ln}(\frac{\kappa }{\eta }+1)+\sqrt{\frac{\kappa }{\eta }}\cdot \arctan (\sqrt{\frac{\eta }{\kappa }})],$$

where k_b_ is Boltzmann’s constant, and *η* is a material-dependent constant related to the bending stiffness of the 2D material.

To better understand the dependences of adhesion induced entropy in 2D materials on adhesion strength and materials-dependent constant *η*, we calculate the entropy variation in adiabatic adhesion processes from MD simulation. The simulation was performed for graphene and *h*-BN on a variety of substrates. Tersoff potential^27^ is used to describe the B-N bond in *h*-BN sheet, and a Lennard-Jones 12-6 potential is used to model the interaction between *h*-BN and the substrates.

**Figure 3:**
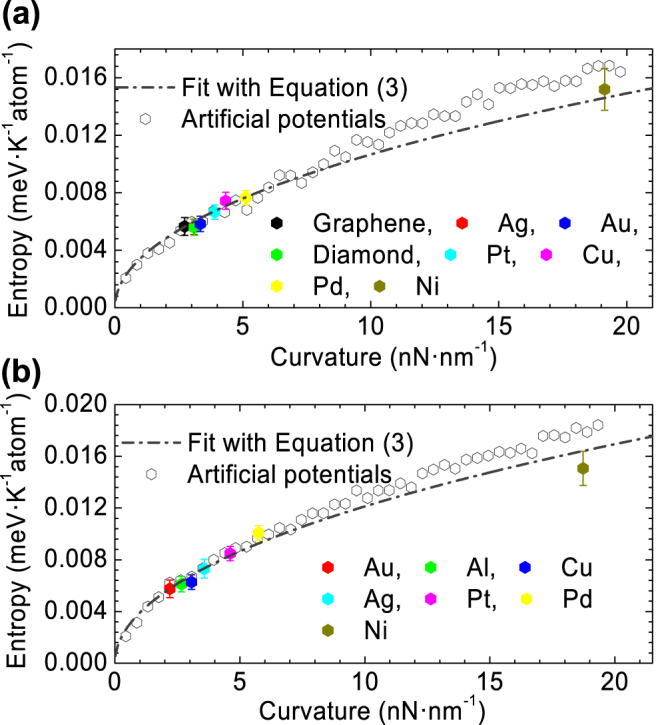
(**a**) Adhesion induced configurational entropy change of graphene on various substrates, including a rigid graphene layer as substrate. The results labelled as “Artificial potentials” are performed on a Pt substrate with artificial potentials to describe the interaction between atoms of the graphene layer and Pt atoms (see Supplemental Materials for details). Equation (3) is fitted to MD results using *η* as the fitting parameter. (**b**) Adhesion induced entropy change of *h*-BN on various substrates.

Figure 3 shows the entropy change as a function of potential curvature *κ*. It is seen that, form both MD simulations (entropy changes are extracted as Δ*S* = −Δ*E*/*T*) and Eq. (3), the adhesion induced entropy change logarithmically increases with the increasing potential curvature *κ*. For physical adhesion through van der Waals forces, Lennard-Jones 12-6 potential is frequently used to describe the interface interactions. In this case, the potential curvature *κ* depends on the potential well *ε* and equilibrium distance *σ*, i.e., *κ* ∝ *εσ* ^−2^. This can explain why the entropy change on nickel (Ni) substrate is much larger than that on others. It is because the adhesion between 2D layers and the Ni substrate is not only stronger in term of adhesion energy but also has a smaller equilibrium distance, which is about 0.21 nm while the equilibrium distances for other substrates are more than 0.3 nm^21, 22^. We note that a chemical adhesion in general induces a stronger interface interaction (with a larger binding energy and a smaller equilibrium distance) due to the creation of chemical bonds, which can consequently generate a larger entropy change.

Equation (3) indicates that the adhesion induced configurational entropy change decreases with an increase in the parameter *η*, which is positively and almost linearly correlated with the bending stiffness of 2D materials (see Supplemental Materials for details). The fitted value of *η* for graphene is about 25% larger than that for *h*-BN (Fig. 3), and the bending stiffness of graphene calculated directly from MD too is 25% larger than that of *h*-BN. Another interesting issue is the adhesion induced entropy change of multiple 2D layers. Multiple layers in general have a larger bending stiffness than a single 2D layer^28^. Therefore, the entropy change in adhesion should be smaller than that of a single layer. Our MD results show that the peeling of a bilayer graphene absorbs the same amount of heat as that for the peeling of a single graphene layer, meaning that the same amount of entropy change is induced in both cases (see Supplemental Material for details). This indicates that the entropy change in the detachment of a bilayer graphene is mostly contributed by the layer in contact with the substrate. Equation (3) also suggests that the heat release or absorption is vanishingly small in adhesion between bulk materials for which the bending stiffness can be considered infinitely large. We therefore conclude that the gas-like adhesion of a 2D material indeed depends on two dominant factors – a low bending stiffness and a large portion of contacting atoms, both of which leads to a significant configurational entropy change during adhesion.

The gas-like adhesion may raise fundamental issues in many applications of 2D materials. For instance, it has been proposed that using shear adhesion force as a retracting force could lead to a ultrahigh frequency for an oscillator composed of a graphene layer and a substrate^17^. Since the adhesion force is temperature dependent, the frequency of this oscillator also may be temperature tunable. On the other hand, the gas-like adhesion also implies a new approach for developing nanoscale actuators that could convert thermal energy into motions. Designing such devices is yet a challenge due to the absence of an efficient energy conversion mechanism at nanoscale. The energy conversion through 2D material attachment/detachment may overcome this challenge due to its thermodynamically reversible feature. In fact, a phenomenon related to this type of energy conversion has already been observed in domino-like collapse and restoration of carbon nanotubes, first in atomistic simulations^12^ and then in experiments^13^ where the energy conversion is based on a change of the cross-section of the carbon nanotube between its usual circular configuration and collapsed flat configuration (in which the shell is partially adhered together). It is found that the circular-to-flat collapse and the flat-to-circular restoration are both temperature dependent. While it has been speculated that this phenomenon is governed by changes in system entropy, our present study on the 2D material adhesion provides a clear theoretical foundation to understand such phenomena.

In summary, we have shown that adhesion between 2D materials and solid substrates is not a pure solid-solid mechanical adhesion but a gas-like adhesion. The attaching/detaching process of a 2D material onto/from a solid surface is an exothermic/endothermic process as the gas adsorption/desorption. The adhesion energy and adhesion forces are not constants solely determined by physical interaction forces. In contrast, they depend also on system temperatures. The underlying physics is that the adhesion generates a confinement on 2D materials and causes a decrease in the configurational entropy. Our findings provide a fundamental understanding of the adhesion of 2D materials.

## Electronic supplementary material

Gas-like adhesion of two-dimensional materials onto solid surfaces: Supplemental materials
