# Supplementary material for: Gas-like adhesion of two-dimensional materials onto solid surfaces
Source: Sci Rep. 2017 Mar 13;7:159. doi: 10.1038/s41598-017-00184-x (PMC5427824; doi:10.1038/s41598-017-00184-x)
Supplement: Supplementary file 1 — Gas-like adhesion of two-dimensional materials onto solid surfaces: Supplemental materials [file 41598_2017_184_MOESM1_ESM.pdf]

Gas-like adhesion of two-dimensional materials onto solid surfaces: Supplmental materials

Zhengrong Guo[1]*, Tienchong Chang[1,2], Xingming Guo[1], Huajian Gao[3]

[1]*Shanghai Institute of Applied Mathematics and Mechanics, Shanghai University, Shanghai Key Laboratory of Mechanics in Energy Engineering, Shanghai 200072, People's Republic of China*

[2]*State Key Laboratory of Ocean Engineering, School of Naval Architecture, Ocean and Civil Engineering, Shanghai Jiao Tong University, Shanghai 200240, People's Republic of China*

[3]*School of Engineering, Brown University, Providence, Rhode Island 02912, USA*

* Corresponding Author(**guozhengrong@shu.edu.cn)**

Molecular dynamic simulation package and interaction potentials

The molecular dynamics (MD) simulations were performed based on the Large-scale Atomic/Molecular Massively Parallel Simulator code (LAMMPS) [1] where the Verlet algorithm with a time step of 1 fs is used to calculate position and velocity for atoms. The second generation reactive empirical bond order (REBO) [2] potential was used to describe the C-C bond interactions in graphene, and the Tersoff potential[3] was used to describe the B-N bond interactions in *h*-BN. These potentials were widely used to investigate the mechanical and thermal properties of the two types of 2D materials. A Lennard-Jones (LJ) 12-6 potential with a cutoff distance of 1 nm was used to describe the grapheme-substrate and *h*-BN-substrate interactions [4]. The values of the LJ parameters ε and σ, obtained by fitting with DFT calculations [4-6], are listed in Table 1. Note that, although the interactions between graphene and some metal substrates may involve hybridizations [5], it had been proved that the LJ potential can capture the adhesive energy as a function of the inter-surface distance quite accurately. In addition, there are two types of atoms in the *h*-BN layer, with uncertain parameter values for the LJ potential. In current simulations, same parameters were used for both types of atoms in *h*-BN. This approximation may have some quantitative effects on the simulations, but should not change our main conclusions.

Table 1. Parameters for the 12-6 Lennard-Jones potential. The parameters were fitted from DFT calculations for Metal/C interaction [3] and Metal/BN [4]. The C^a/C is for graphene/graphene interaction, and C^b/C for graphene/diamond interaction [5], and κ is the curvature at the bottom well of the LJ potential.

**Graphene**	Ni/C	Cu/C	Au/C	Pt/C	Ag/C	Pd/C	C^a/C	C^b/C
ε (meV)	39.50	10.40	8.60	9.09	7.95	12.0	2.97	3.15
σ (nm)	0.210	0.298	0.330	0.330	0.326	0.295	0.3407	0.342
κ (N/m)	19.136	4.334	3.396	3.903	3.094	5.120	2.724	3.107
***h*-BN**	Ni/BN	Cu/BN	Au/BN	Pt/BN	Ag/BN	Pd/BN	Al/BN	
ε (meV)	31.31	7.50	5.53	10.5	8.91	13.12	6.15	
σ (nm)	0.206	0.332	0.328	0.331	0.341	0.305	0.372	
κ (N/m)	18.727	3.069	2.20	4.613	3.576	5.751	2.668	

Attachment and detachment of the graphene ribbon on/off a substrate

The size of the Pt substrate used in the first two simulations is 41 nm (length) × 9.7 nm (width) × 4 nm (height), while the size of graphene ribbon is 28.5 nm (length) × 9.7 nm (width). Periodical boundary condition is applied in the width direction.

In the peeling simulation (FIG. 1b), a graphene ribbon is initially placed on the (111) surface of the Pt substrate with its edge being 0.95 nm from the left sidewall of the substrate. Before we perform the simulations under an adiabatic condition (using an NVE ensemble), we first relax the graphene for 200 ps at 300K using an NPT ensemble to remove any possible pressure in the width direction. Because the relaxation is performed only for graphene ribbon, the substrate may be slightly stretched or compressed in the width direction. The temperature of graphene at time t is obtained by averaging the instantaneous temperatures through a small time period of 1 ps around the time t.

The peeling and attaching speeds are 10 m/s in all our simulations. To check whether the peeling speed has an effect on the energy conversion, we performed the detaching simulations with different peeling speeds ranging from 10 m/s to 50 m/s. It is seen that the peeling speed slightly affects the temperature change during the detachment. This effect may be caused by the interface friction which is dependent on sliding velocity at nanoscale.

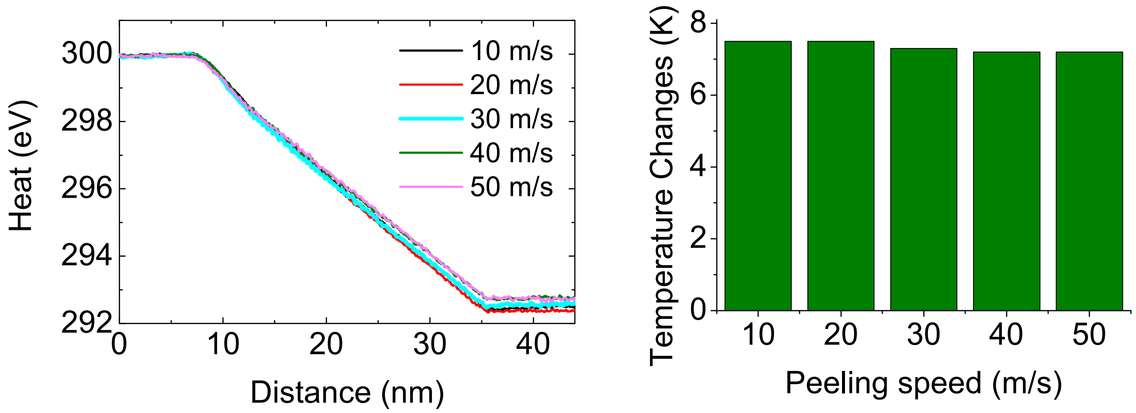

Figure S1. Temperature change of a graphene ribbon during the adiabatic processes of zero-degree peeling (left). The temperature changes versus peeling speeds (right).

Figure S2 shows the temperature distribution along the length of the graphene ribbon during the detachment. The graphene ribbon is divided into 12 regions along its length direction for calculating the local temperature of different each regions. It is seen that the temperatures of different regions are almost the same at each moment during the whole detachment. This is because the thermal conductivity of graphene is

extremely high and each state in the peeling process is a quasi-static state.

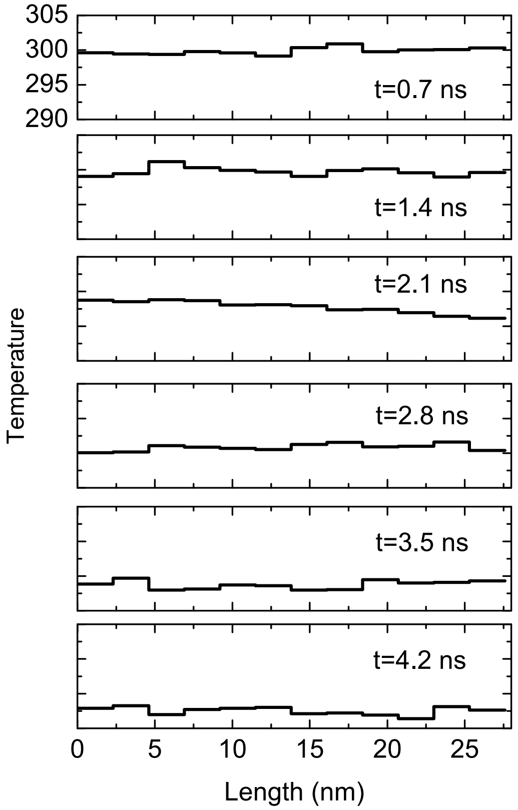

Figure S2. Temperature distribution of the graphene ribbon along its length direction during a peeling process.

Statistics of the peeling force and adhesion heat (entropy)

We calculated the peeling force and the adhesion heat in the same way described in section S-1, except that the NVT ensemble is used to keep the system at constant temperatures. The calculated shear adhesion force and adhesion induced heat change fluctuate with time, and are Gaussian-distributed, as shown in FIG. 2c-d. By fitting their distributions to Gaussian functions, we obtained the average values for the shear adhesion and adhesion induced heat. The existence of friction may slightly bias the adhesion heat and peeling force we measured. The measured heat in a peeling process is $\Delta E^{s}_{\text{measure}} = \Delta E - \Delta E_{\text{Friction}}$, while the measured heat in a stamping process is $\Delta E^{a}_{\text{measure}} = \Delta E + \Delta E_{\text{Friction}}$. Thus the adhesive heat without frictional bias can be obtained by $\Delta E = (\Delta E^{a}_{\text{measure}} + \Delta E^{s}_{\text{measure}})/2$. The shear adhesion forces is obtained in the same way.

Simulation of peeling on a nonrigid substrate

In all our simulations, the atoms in the substrates were fixed to eliminate fluctuations of the calculated results. To assure this setting has no significant influence on our conclusion, we performed simulations of a graphene ribbon supported by a Pt substrate with its atoms freely vibrated. In this case, we found that the adhesive heat is difficult to be measured because of the relatively very large fluctuations due to the large amount of the substrate atoms. Fortunately, the peeling force on the graphene ribbon can still be calculated as shown in FIG. S1. However, the entropic part of the peeling force in this case is about 50% larger than that on the rigid substrate. This may be attributed to two possible mechanisms. First, the thermal vibration of substrate can induce fluctuations of the out-of-plane motion of 2D layers, which may increase the adhesion entropy. Second, the quasi-2D structure of the substrate experiences significant out-of-plane thermal vibrations, which could enhance the adhesion caused entropy too. Moreover, the substrate is stacked up by layers of atoms arranged in (111) planes. Although they are chemically bonded together, their out-of-plane vibration still exist, especially for the most top surface layer. However, a theoretical estimate of the contribution of these mechanisms is difficult and material-dependent, thus the present study focus only on the adhesion entropy contributed by the 2D layers.

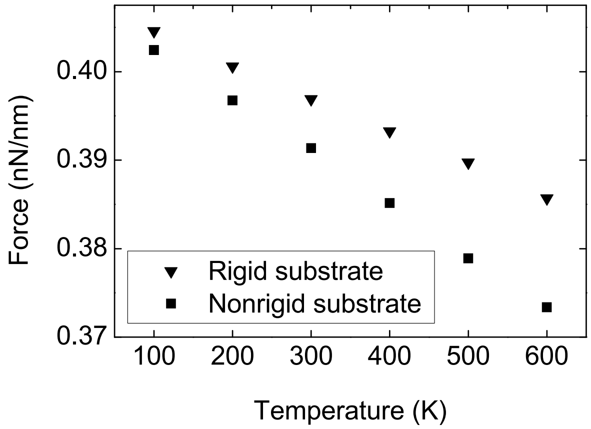

FIG. S3. Comparison of the peeling force on the rigid and nonrigid substrates.

We further performed an adiabatic simulation of a peeling/attaching cycle of graphene onto a nonrigid Pt substrates. Figure S4 shows the temperature change of the system during the peeling and attaching processes. The amount of converted energy estimated from temperature change is 50% larger than that on the rigid substrate, which is consistent with Figure S3. Note that change the substrate from a rigid substrate to a nonrigid one does not affect the reversibility of the two processes. The dissipative heat is estimated about 6.7% (derived by dividing the dissipative temperature change by double of the total temperature change), which is 8% on a rigid substrate.

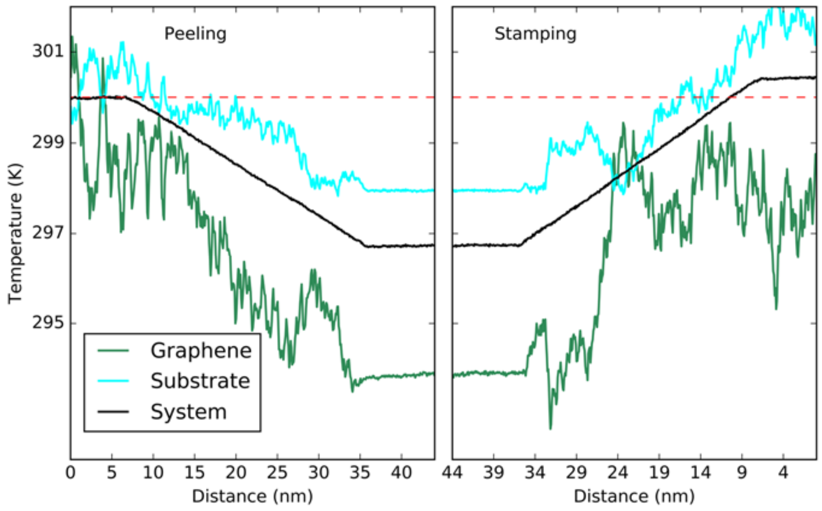

Figure S4. Temperature change in peeling and stamping processes of a graphene layer onto and off a nonrigid substrate.

Simulations with artificial potentials between 2D layers and substrates

To capture the dependence of adhesion induced entropy variation on curvature κ directly from MD simulations, we simulate the 2D layer on a Pt substrate with the artificial potential parameters for layer/substrate interaction; a constant equilibrium distance of 0.33 nm and a varying well-depth from 1 meV to 50 meV, for yielding different curvature κ (Figure 3).

Simulations of peeling/attaching processes of a bilayer graphene onto a Pt substrate

The temperature change of a bilayer graphene ribbon in adiabatic peeling/attaching is about 4 K, which is 8.2 K for a single layer graphene in the adiabatic peeling/attaching. In other words, a bilayer graphene has the same converted energy in adhesion with a single layer, but only half of the per-atom value of converted energy and entropy change. This indicates that the energy conversion is mostly contributed by the layer that in contact with the substrate.

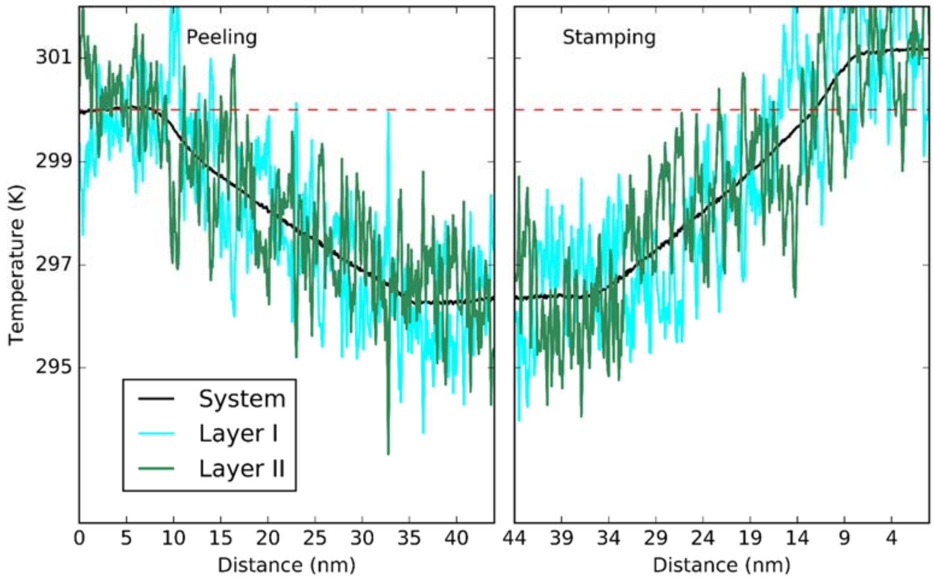

Figure S5. Temperature change of a bilayer graphene ribbon during the adiabatic processes of zero-degree peeling/attaching from a Pt substrate.

Derivation on the adhesion induced entropy reduction

Here we consider the simplest situation that a 2D crystal with N lattices, and each lattice cell contains only one atom with mass m. The expression of per atom entropy of this system can be given by

$$S = \frac{k_B}{N} \ln \Omega \tag{S1}$$

where k_B is Boltzmann constant, Ω is the total number of the microstates. Only those microstates related to out-of-plane normal modes (phonon) are considered. The number of microstates for a normal mode with frequency ω_i is given by

$$\Omega(\omega_i) = \frac{1}{e^{\hbar\omega_i/k_B T} - 1} \approx \frac{k_B T}{\hbar\omega_i} \tag{S2}$$

where T is the system temperature, $\hbar$ is the Planck constant. The approximation in equation (S2) breakdown only when the temperature becomes extremely low. The total number of microstates of all the out-of-plane normal modes is

$$\Omega = \prod_{i=1}^{N} (k_B T / \hbar\omega_i) \tag{S3}$$

If harmonic approximation is adopted for describing the influence of adhesion on the normal vibration, the frequency of the out-of-plane normal modes is shifted to

$$\omega' = \sqrt{\omega + \kappa / m} \tag{S4}$$

where κ is the force constant of the harmonic potential (for a general potential, it is the curvature of the potential at bottom). With those equations, the per atom adhesion entropy between freestanding state and adhered state can be written as

$$S - S^{\dagger} = \frac{1}{N} k_B \sum_{i=1}^{N} \ln \sqrt{1 + \kappa / m\omega_i^2} \tag{S5}$$

The equation (S5) can be recast in an integral form

$$S - S^{\dagger} = \frac{1}{N} k_B \int_0^{\omega_{max}} \rho(\omega) \cdot \ln \sqrt{1 + \kappa / m\omega^2} \, d\omega \tag{S6}$$

where $\rho(\omega)$ is the distribution of the density of normal modes (DOS). It can be seen from Equation (S5) that the entropy change is mainly dependent on those modes with low frequency. In theoretical studies, a distribution of $\rho(\omega) = c\omega^{\tau}$ (where c and τ are constants) is usually used for those normal modes. For 2D materials, the distribution of out-of-plane modes is $\rho(\omega) = c$. A maximal frequency ω_{max} can be defined for the existence range of frequency. Its relation with the total number of modes is given by $N = \rho(\omega)\omega_{max}$. In fact, the maximum frequency ω_{max} is a material-dependent constant, which is linearly dependent on the dihedral force constant, while the bending rigid of 2D materials G is also linear dependent on dihedral constant. Thus we expect $\omega^2_{max} \propto G$.

Finally, the adhesive entropy is given by

$$S - S^{\dagger} = \frac{1}{2} k_{\mathrm{B}} \left\{ \ln\left(\frac{\kappa}{\eta} + 1\right) + \sqrt{\frac{\kappa}{\eta}} \cdot \mathrm{Arctan}\left(\sqrt{\frac{\eta}{\kappa}}\right) \right\} \qquad \text{(S6)}$$

where $\eta = m\omega_{\max}^{2}$ is also a material-dependent constant.

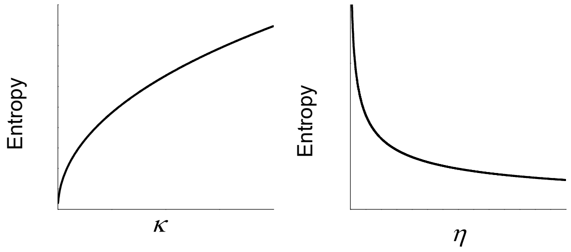

FIG. S6. Adhesion induced entropy change versus curvature κ and material-dependent parameter η.

The derivation above can be easily extended to the situation that each lattice cell contains two atoms. For graphene, because the two atoms in each unit cell have the same atoms, thus the equations from (S1) to (S6) have exactly the same forms. For *h*-BN, the only difference is the mass and it is given by $m_{\mathrm{BN}} = 2m_{\mathrm{B}}m_{\mathrm{N}} / (m_{\mathrm{B}} + m_{\mathrm{N}})$, where the m_{B} and m_{N} are the mass of boron atom and nitride atom, respectively.

References

[1] S. Plimpton, J. Compu. Phys. **117**, 1 (1995).

[2] D. W. Brenner, O. A. Shenderova, J. A. Harrison, S. J. Stuart, B. Ni, and S. B. Sinnott, J. Phys-Condens. Mat. **14**, 783 (2002).

[3] A. Kınacı, J. B. Haskins, C. Sevik, and T. Çağın, Phys. Rev. B **86**, 115410 (2012).

[4] M. Bokdam, G. Brocks, M. I. Katsnelson, and P. J. Kelly, Phys. Rev. B **90**, 085415 (2014).

[5] I. Hamada and M. Otani, Phys. Rev. B **82**, 153412 (2010).

[6] W. Hu, Z. Li, and J. Yang, J. Chem. Phys. **138**, 054701 (2013).
